# Quercetin affects uterine smooth muscle contractile activity in gilts

**DOI:** 10.1371/journal.pone.0252438

**Published:** 2021-07-16

**Authors:** Aleksandra Zygmuntowicz, Włodzimierz Markiewicz, Tomasz Grabowski, Artur Burmańczuk, Alla Vyniarska, Jerzy Jan Jaroszewski

**Affiliations:** 1 Department of Pharmacology and Toxicology, Faculty of Veterinary Medicine, University of Warmia and Mazury in Olsztyn, Olsztyn, Poland; 2 Polpharma Biologics SA, Gdańsk, Poland; 3 Department of Pharmacology Toxicology and Environmental Protection, Faculty of Veterinary Medicine, University of Life Sciences, Lublin, Poland; 4 Department of Pharmacology and Toxicology, Stepan Gzhytskyi National University of Veterinary Medicine and Biotechnologies, Lviv, Ukraine; Max Delbruck Centrum fur Molekulare Medizin Berlin Buch, GERMANY

## Abstract

Quercetin is a polyphenolic flavonoid occurring in leaves, stems, flowers and fruits of many plants. In traditional Chinese medicine, it is used as a natural therapeutic agent with a broad spectrum of activities (antioxidant, neuroprotective, anti-inflammatory, anticancer, antibacterial and antiviral). Moreover, quercetin affects function of the reproductive tract, however the knowledge of this activity is still fragmentary. Therefore, this study aimed to determine the influence of quercetin on the contractile activity of the porcine myometrium collected from immature (n = 6), cyclic (n = 6) and early pregnant (n = 6) gilts. Strips of the myometrium (comprising longitudinal and circular layer) were resected from the middle part of the uterine horns and the isometric contractions were recorded. After 60–90 min of preincubation, the strips were stimulated with quercetin in increasing (10^−13^–10^−1^ M) concentrations and the changes in the tension amplitude and frequency of contractions were measured. Quercetin decreased (P<0.01–0.001) the amplitude of contractions at concentrations 10^−11^–10^−1^ M and 10^−10^–10^−1^ M in cyclic and early pregnant groups, respectively. The frequency of contractions decreased in all groups but was the highest (at concentrations 10^−11^–10^−1^ M; P<0.05–0.001) in the cyclic group and the lowest (at concentrations 10^−5^–10^−1^ M; P<0.01) in the immature group. The tension decreased only in the cyclic group after quercetin administration in high concentrations (10^−6^–10^−1^ M; P<0.05–0.01). The results indicate that quercetin causes relaxation of the porcine uterine smooth muscle but this activity is strongly related to the physiological status of the gilts.

## Introduction

Quercetin is one of the natural flavonoids occurring in fruits (e.g. apple, blueberries, black currant, orange), vegetables (e.g. onion, broccoli, spinach, cabbage), flowers (e.g. hawthorn, chestnut, black lilac) and herbaceous plants (e.g. horsetail, rue, chamomile) [1]. It is also present in tea, wine [2] and honey [3].

After oral administration, quercetin is deglycosylated in the lumen of the small intestine in the brush border membrane by lactose-frisine hydrolase, from where the detached aglycons are absorbed by the intestinal wall through passive diffusion. Quercetin is also deglycosylated in enterocytes, from where the aglycones are absorbed and metabolized in the small intestine, colon, liver and kidneys through active transport by the Na + / glucose transporter. Subsequently, in liver epithelial cells, quercetin is metabolized by phase II metabolism proteins. The resulting metabolites are then transported through the portal vein to the liver where they are further degraded. Metabolites formed in the liver travel with the blood to the tissues, where they show different biological activity. In turn, metabolites resistant to the action of intestinal hydrolases and not absorbed in the small intestine pass into the large intestine, where they are transformed with the participation of enzymes secreted by enterobacteria [4].

Quercetin has strong antioxidant properties because it has the ability to scavenge free radicals and chelating transition metal ions [5]. It is one of the most potent scavengers of reactive oxygen species [6]. Quercetin not only prevents the propagation of lipid peroxidation but also increases glutathione levels which contribute to preventing free radical formation [7]. The antioxidant activity of quercetin includes the inhibition of lipid peroxidation of cell membranes, protects against the oxidation of low-density lipoproteins (LDL) and increases high-density lipoproteins [8]. Because of antioxidant properties, quercetin can reduce the occurrence of neurodegenerative processes occurring, e.g. in Alzheimer’s and Parkinson’s diseases [9]. Moreover, the consumption of flavonoids showed a reduction in coronary heart disease [10]. *In vitro* studies have been demonstrated that quercetin exerts endothelium-independent vasodilator effects, protective effect on nitric oxide and endothelial function under conditions of oxidative stress, platelet antiaggregant effects, inhibition of LDL oxidation, reduction of adhesion molecules and other inflammatory markers and prevention of neuronal oxidative and inflammatory damage [11]. Moreover, it has anti-cancer properties through its antioxidative effects as well as kinase and cell cycle inhibition and induced apoptosis [12]. However, the pro-oxidant activity of quercetin has also been observed [13].

The available research results also indicate that quercetin significantly affect reproductive tract function. Both *in vitro* and *in vivo* results demonstrated by Park et al. [14] suggest that quercetin can be used as a natural therapeutic to reduce and/or treat endometriosis. Moreover, quercetin improved polycystic ovary syndrome in rats through its phytoestrogenic effects and mimicking oestrogen’s function, expressed as an increase in the adiponectin level and a decrease in both the expression of aromatase and the oestradiol level [15]. It has also been demonstrated that the addition of quercetin to feed significantly increased the secretion of oestradiol, progesterone, follicle-stimulating hormone and luteinizing hormone in laying hens [16].

It has been demonstrated that quercetin has an anti-inflammatory activity which is associated with inhibition of arachidonic acid metabolism and production of inflammatory mediators such as prostaglandins and leukotriens [17]. However, it is well-documented that both prostaglandins and leukotrienes are involved in the regulation of uterine contractile activity [18–20]. Moreover, it was shown that quercetin relaxes the tonic contraction in the rat uterus induced by prostaglandin F_2α_ (PGF_2α_), oxytocin (OT) and carbachol [21].

Quercetin is a naturally occurring selective phosphodiesterase-4 (PDE_4_) inhibitor [22]. It has been demonstrated that two PDE_4_ inhibitors (citomilast and rolipram) inhibit smooth muscle contractions in the human uterus and these changes were strongly expressed in pregnant than nonpregnant myometrium [23]. Moreover, it has also been shown that phosphodiesterase activity increased from proestrus to a peak in late metestrus and then decreased until the next proestrus period during the oestrous cycle in rats [24].

At present, there are no data on the role of quercetin in the regulation of the uterine contractile activity in pigs. Therefore, this study aimed to determine the effect of quercetin on the contractility of the myometrium in females with different physiological status, i.e. immature, cyclic and early pregnant gilts.

## Material and methods

### Reagents

All the inorganic salts (NaCl, KCl, CaCl_2_, MgCl_2_, NaHCO_3_) needed to prepare the Krebs-Ringer buffer, ethanol and glucose were purchased from Chempur (Piekary Śląskie, Poland). Acetylcholine chloride (ACh) and quercetin (Que) were purchased from Sigma (St. Louis, MO, USA). A stock solution of quercetin (10^−1^ M) was prepared in ethanol and series of dilutions were made with deionized water on the day of experimentation.

## Animals

Eighteen crossbreed gilts (Large White x Polish Landrace) came from private breeding farm (L. Wisniewski breeding farm, Krolikowo, Poland) were assigned into three experimental groups (n = 6 in each). In the first group (immature), the uteri of gilts at the age of 4 to 5 months with an average body weight of 50–55 kg were used. In the second group (cyclic), the uteri of gilts on days 12–14 of the oestrous cycle at the age of 7 to 8 months with an average body weight of 120–130 kg were collected; the phase of the oestrous cycle was confirmed based on the morphology of the ovaries [25]. In the third group (pregnant), the uteri were collected from gilts on days 12–16 of gestation (implantation period); about 7–8 months old, weighing 120–130 kg; pregnancy was confirmed by washing the horns of the uterus with 10 mL PBS, pH = 7.4 in the presence of embryos. The procedure of selection and insemination of the gilts in this group was described previously [26]. Uteri from all animals were collected immediately after slaughter in meat processing plant ("TOMUS" Tomasz Reihs, Krolikowo, Poland) and then transported on ice to the laboratory within 0.5 h.

All studies were conducted in accordance with the ethical standards of the Local Ethics Committee of the University of Warmia and Mazury in Olsztyn, which approved all procedures and granted consent (No. 91/2011 / DTN).

### Preparation of uterine strips and measurement of their contraction

The uterine strips used in the study were prepared as previously described [27, 28]. Briefly, after slaughter uteri were placed on ice and strips (3 × 5 mm) of myometrium (comprising longitudinal and circular layer) were collected from the middle part of the horns. Thereafter, the strips were washed in saline and mounted between two stainless steel hooks in an organ bath (Schuler Organ bath type 809; Hugo Sachs Electronic, Germany) under a resting tension of 10 mN. The strips were kept in 5 mL of the Krebs-Ringer solution with the following composition (mM/l): NaCl—120.3, KCl—5.9, CaCl_2_−2.5, MgCl_2_−1.2, NaHCO_3_−15.5, glucose—11.5, temp. 37°C and pH 7.4. During the experiment, the solution was continuously saturated with a mixture of 95% O_2_ and 5% CO_2_. For measuring the contractile activity of myometrial strips, a force transducer (HSE F-30 type 372) with a type 570 bridge coupler was used. A graphic recording was made on a recorder (Hugo Sachs Elektronik) with the HSE-ACAD/W software. The recording was started after a 60–90 min equilibration period. To check the viability of tissues and their usefulness for the study the strips were stimulated with increasing (10^−6^–10^−4^ M) concentrations of ACh [29]. After washing, the strips were incubated with increasing (10^−13^–10^−1^ M) concentrations of quercetin (Que) added at 15-minute intervals. Thereafter, the organ bath was washed and ACh at concentrations of 10^−6^–10^−4^ M was administered. Only those results were differences in response to ACh stimulation at the beginning and the end of the experiment were less than 20% were included in the statistical analysis [29].

### Pharmacodynamic analysis

A sigmoid dose-response model relationship between examined effects (amplitude, frequency and tension) and quercetin concentration was analysed by nonlinear regression analysis with automatic outlier elimination (ROUT method, Q = 1%). All calculations were performed using GraphPad Prism version 6.07 (Graphpad Software, San Diego, CA, USA). In the first phase, exploratory dose-response analyses were conducted using various models. Finally, a log (inhibitor) vs. response model (variable-slope) model without constants was selected. The selected parameters were calculated: E_max_−maximal effect value, E_0_ –no effect value (baseline), Slope–value of Hill slope of the model, and LogIC_50_ –log-transformed concentration of quercetin that gives a half-maximal response.

### Statistical analysis

Tension (resting/baseline tension expressed in mN), frequency (the number of observed peaks) and amplitude (the difference between the minimum and maximum value for a single contraction expressed in mN) as parameters of spontaneous myometrial strips contractile activity were calculated for 10 min before quercetin administration (pre-treatment period) and accepted as 100%. The results calculated for 10 min periods after the administration of each concentration of quercetin were expressed as a percentage of the tension, frequency and amplitude measured in the pre-treatment period. The statistical significance of the differences between pre-treatment and post-treatment periods as well as between three examined groups of animals were assessed by one-way analysis of variance ANOVA (Graphpad Prism 6.07, Graphpad Software, San Diego, CA, USA) followed by Bonferroni’s multiple comparison test. The differences with a P-value less than 0.05 were considered significant.

A separate statistical analysis was performed to assess the quality of pharmacodynamic models. The models were qualified for raw data analysis based on Akaike information criterion (AIC) and after evaluation, the standard error of the estimate by robust standard deviation of the residuals, calculation (RSDR) of the best model was selected.

## Results

**Fig 1:**
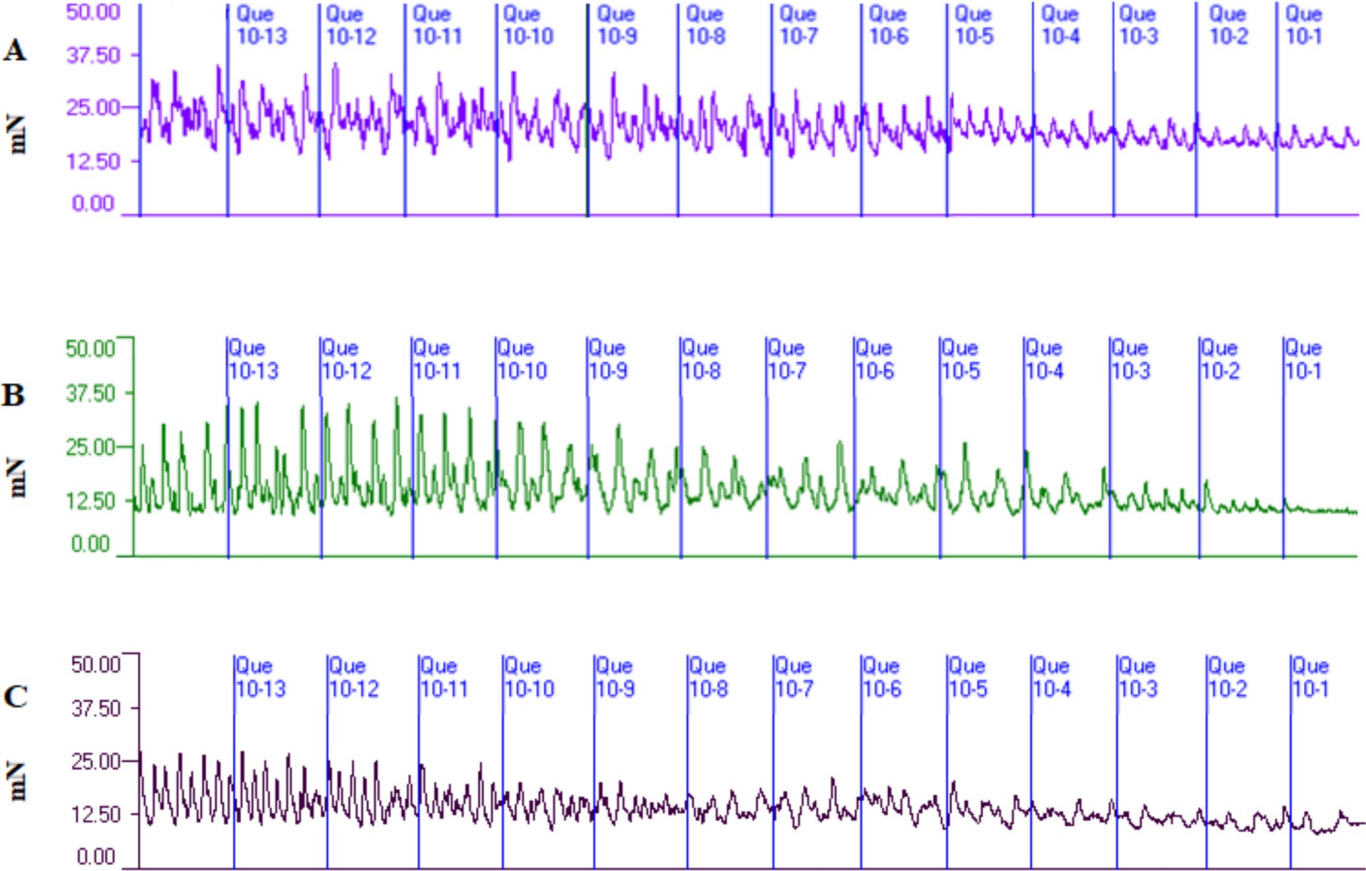
Diagrams of contractile activity. Diagrams showing the contractile activity of the porcine myometrial strips collected from sexually immature gilts (A), on days 12–14 of the oestrous cycle (B) and on days 12–16 (C) before (15 min) and after administration of concentration (Que; 10^−13^–10^−1^ M).

**Fig 2:**
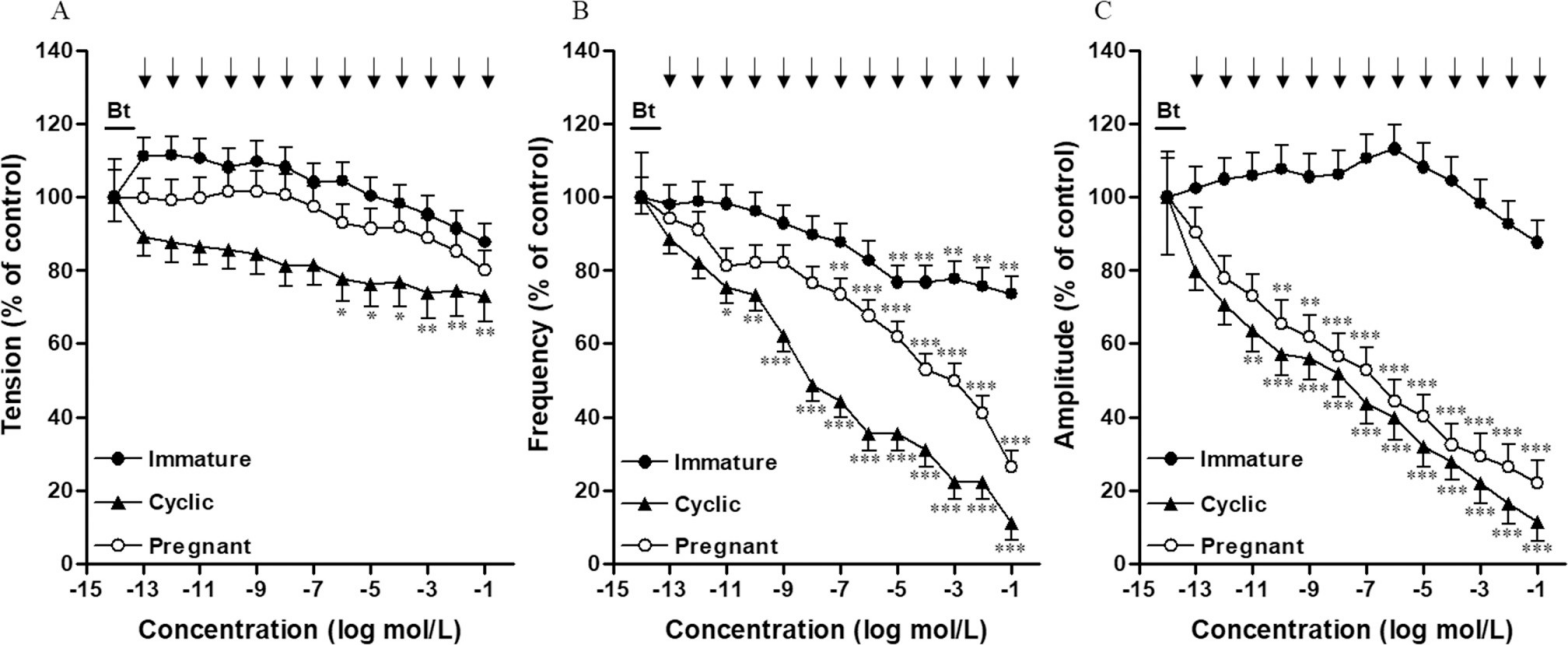
Effect of Que on uterine contractions. Effect of quercetin (Que) on tension (A), frequency (B) and amplitude (C) of contractions of myometrium in immature pigs (-●-; n = 6) and on days 12–14 of the oestrous cycle (-▲-; n = 6) and 12–16 of pregnancy (-○-; n = 6). The results calculated for a 15 min period after treatment with each quercetin concentration were expressed as a percentage (mean ± SD) of the amplitude, frequency and tension determined for a 15 min period before treatment (Bt). *p<0.05, **p<0.01,***p<0.001 –significant differences as compared to the contractile activity before the treatment.

Typical diagrams showing contractile activity before and after quercetin administration in all examined groups are presented in Fig 1. Quercetin significantly (P<0.05–0.01) decreased the tension only in the cyclic group at concentrations of 10^−6^–10^−1^ M as compared to the pre-treatment period but it did not alter this parameter in immature or early pregnant groups (Fig 2). The frequency of contractions decreased in all examined groups and these changes were significant after quercetin administration in concentrations of 10^−5^–10^−1^ M (P<0.01) in the immature group, in concentrations of 10^−11^–10^−1^ M (P<0.05–0.001) in the cyclic group and in concentrations of 10^−7^–10^−1^ M (P<0.01–0.001) in the early pregnant group compared to the pre-treatment period (Fig 2). Quercetin significantly (P<0.01–0.001) inhibited the amplitude of contractions in concentrations of 10^−11^–10^−1^ M in the cyclic group and in concentrations of 10^−10^–10^−1^ M the early pregnant group but did not cause significant changes in the immature group compared to the pre-treatment period (Fig 2).

Calculated pharmacodynamic parameters and statistical parameters describing PD model goodness-of-fit are presented in Table 1. In all groups (immature, cyclic, pregnant), the effect of quercetin on model parameters was noted. Both in case tension and frequency, the LogIC_50_ values were highest for the immature group and the lowest for cyclic (LogIC_50_ value: cyclic<pregnant<immature). Regarding amplitude, the LogIC_50_ values were highest in the immature group and lowest for pregnant pigs (LogIC_50_ value: pregnant<cyclic<immature).

**Table 1:** The pharmacodynamic of Que.

Parameters	Tension			Frequency			Amplitude		
	Immature	Cyclic	Pregnant	Immature	Cyclic	Pregnant	Immature	Cyclic	Pregnant
E_max_	9.66	14.54	11.49	23.70	10.43	5.151	14.75	16.12	25.03
	[8.66–10.65]	[13.10–15.99]	[10.65–12.33]	[23.28–24.12]	[1.83–19.03]	[3.74–6.56]	[12.62–16.89]	[7.64–24.60]	[-8.70–58.75]
E_0_	11.32	12.75	9.81	19.65	-0.877	3.06	16.96	5.26	4.89
	[10.37–12.27]	[11.79–13.70]	[9.02–10.60]	[19.12–20.19]	[-4.62–2.86]	[1.98–4.15]	[15.82–18.11]	[2.49–8.02]	[-1.46–11.25]
Slope	-0.38	0.57	0.62	0.39	0.08	0.33	-1.13	0.22	0.13
	[-1.46–0.70]	[-1.67–2.81]	[-1.29–2.53]	[0.17–0.62]	[-0.01–0.18]	[-0.56–1.23]	[-7.46–5.20]	[-0.11–0.55]	[-0.11–0.37]
LogIC_50_	-6.356	-8.177	-6.792	-6.33	-9.380	-7.245	-3.306	-8.987	-10.50
	[-9.88–2.82]	[-11.61–4.74]	[-9.27–4.31]	[-6.99–5.666]	[-14.48- -4.27]	[-10.94–3.54]	[-5.99–0.617]	[-12.65–5.32]	[-20.90–0.090]
AIC	151.4	210.9	161	-9.92	-23.01	13.02	275.4	276	251.4
RSDR	2.37	3.37	2.51	0.946	0.9845	0.326	4.95	5.17	4.62

Dose-response value (shown as mean with 90% confidence interval) of calculated parameters based on changes in tension and amplitude and frequency of contractions of the uterine myometrial strips collected from gilts immature (n = 6), on days 12–14 of the oestrous cycle (cyclic; n = 6) and 12–16 of pregnancy (pregnant; n = 6) and treated with increasing (10^−13^–10^−1^ M) concentrations of quercetin (Que).

E_max_−maximal effect value, E_0_ –no effect value (baseline), Slope–value of Hill’s slope of the model, LogIC_50_ –log-transformed concentration of quercetin that gives half-maximal response, AIC–Akaike information criterion, RSDR–robust standard deviation of the residuals.

## Discussion

Despite a large number of studies on the biological activity of quercetin and its derivatives, these activities have still not fully been described. Quercetin widely exists in the human’s and animals daily food. Therefore, it is important to determine its influence on human and animals health. One of the interesting activities of quercetin is the ability to affect the contractility of the smooth muscle of the porcine uterus. Our results indicate that quercetin in the greatest extent reduced contractile activity in the uterine strips collected from the cyclic animals. In the case of the amplitude and frequency of contractions, these changes were also strongly expressed in the uteri of early pregnant gilts, while in the uteri of immature animals significant changes concerned only the reduction of the frequency of contractions and after application of quercetin in high concentrations. Stronger relaxant effect observed in the cyclic and early pregnant gilts compared to the immature animals was also confirmed by pharmacodynamic analysis. This indicates that the relaxing effect of quercetin is dependent on the physiological status and is different in sexually immature pigs compared to animals with fully-developed neuroendocrine regulation.

The mechanism of relaxation caused by quercetin in the porcine uterus is unknown. The available research results indicate that the uterine contractile activity in gilts is the result of multiple mechanisms, including adrenergic [29, 30], cholinergic [29, 31] and hormonal [32, 33] regulation. It has been documented that the prevalence of ß-adrenergic over α-adrenergic receptors is responsible for the inhibition of uterine contractile activity and that the level of ovarian steroid hormones affects the concentration and distribution of these receptors [31, 34]. In the current study, quercetin caused significant relaxation on days 12–14 days of the oestrous cycle and in early pregnancy and these changes were similar to those observed after β_2_-and β_3_-adrenergic receptor stimulation in the same examined periods [29, 34]. This suggests that ß-adrenergic regulation may be involved in porcine uterine relaxation after quercetin treatment, although it needs further study to determine how this mechanism should be examined.

The relaxation caused by quercetin may be also a result of inhibition of the mechanisms responsible for the contraction of the myometrium. In the uterus of pigs, contractions can be stimulated by acetylcholine (ACh) [35], oxytocin (OT) [33, 36] or prostaglandins [18, 33, 36]. It has been documented that ACh causes contraction of the myometrium through the activation of the muscarinic M_3_ receptor [35]. In the authors’ previous study, ACh increased tension and frequency of contractions and decreased the amplitude of contractions in both the cyclic (12–14 days of the oestrous cycle) and early pregnant (12–14 days of gestation) uterus but these changes were more evident in pregnant (especially with the presence of embryos uterus) than cyclic animals [29]. In the present study, quercetin strongly inhibited the frequency and amplitude of contractions and, to a small extent, the tension, and these changes were more evident in cyclic pigs. Thus, if the changes after ACh administration are greater in early pregnant than cyclic uteri, the diastolic effect following the administration of quercetin may be more visible in cyclic animals, as was observed in the present study.

It is well documented that uterine contractile activity is controlled by OT and prostaglandins [29, 33, 35]. Oxytocin receptors are present in the porcine uterus and their stimulation affects uterine contractile activity [33]. Moreover, it is assumed that OT, by interaction with their receptors, is involved in the control of PGF_2α_ and prostaglandin E_2_ (PGE_2_) secretion in pigs [37, 38]. The number of OT receptors on days 14–16 of the oestrous cycle and gestation is similar in the myometrium but much higher in the endometrium [39]. It was also shown that basal and OT-induced mobilisation of Ca^2+^ was higher in myometrial cells recorded on days 14–16 of the oestrous cycle compared to data from the same days of pregnancy and, following pre-incubation of myocytes with progesterone, the effect of OT on Ca^2+^ was delayed and inhibited in uterine tissue collected from cyclic and early pregnant animals, respectively [37]. However, in the presence of progesterone, no stimulating effect of OT on the secretion of PGF_2α_ and PGE_2_ was observed [39]. Kurowicka et al. [31] demonstrated that OT stimulated the contractile activity of the uterine muscles in early pregnancy and was inhibited by progesterone but such influence was not observed during the oestrous cycle. Progesterone suppresses uterine contractility and it is suggested that this effect is non-genomic and due to the inhibition of OT action on its receptors [40].

It has been documented that influence of prostanoids is regulated by the contractile (FP, EP_1_, EP_3_) and relaxatory (DP, IP, EP_2_) receptors present in the porcine uterine smooth muscle [41]. In the authors’ previous study, it was shown that PGE_2α_ increased tension and amplitude of contractions and decreased the frequency of contractions in porcine uterine tissue, mainly in the presence of embryos but such evident changes were not observed after PGE_2_ administration [29]. It has been also shown, that in the inflamed porcine uterus, PGE_2_ decreased contraction intensity (acting through EP_2_ and EP_4_ receptors) [18] while prostacyclin increased contractility [28], which suggests that the inflammatory process changes the response of prostanoid receptor stimulation. Catarino et al. [17] suggested that anti-inflamatory activity of quercetin is associated with the inhibition of arachidonic acid metabolism and production of inflammatory mediators such as prostaglandins and leukotriens. Taking into account the above, it is assumed that the relaxant activity of quercetin in the porcine uterus may be a result of many mechanisms responsible for the regulation of uterine contractility.

It has been suggested that quercetin inhibits phosphodiesterase-4 (PDE_4_) activity [22]. Oger et al. [23] showed that citomilast and rolipram (PDE_4_ inhibitors) inhibit smooth muscle contractions in the human uterus and these changes were strongly expressed in pregnant than nonpregnant myometrium. The relaxative effect of rolipram was also observed in myometrium collected from women undergoing caesarian section in weeks 38–42 [42] and 37–42 [43] of pregnancy. It has been also shown that roliplam was more effective than theophylline (non-selective PDE inhibitor) in inhibiting the OT-induced contractions in the human uterus [44]. Although this suggests that this mechanism may also be responsible for the relaxative effect of quercetin in the current study, it should be confirmed in an experimental study.

## Conclusions

In conclusion, the present study shows that quercetin causes relaxation of the myometrium in both cyclic and early pregnant pigs under progesterone dominance. However, the exact mechanism of this action has yet to be determined.
